# Autoimmune reaction against pancreatic beta cells in children and adolescents with simple obesity

**DOI:** 10.3389/fendo.2022.1061671

**Published:** 2022-12-14

**Authors:** Aneta Chylińska-Frątczak, Iwona Pietrzak, Arkadiusz Michalak, Krystyna Wyka, Agnieszka Szadkowska

**Affiliations:** ^1^ Department of Pediatrics, Endocrinology, Diabetology and Nephrology, Maria Konopnicka University Pediatrics Center, Lodz, Poland; ^2^ Department of Pediatrics, Diabetology, Endocrinology and Nephrology, Medical University of Lodz, Lodz, Poland; ^3^ Department of Biostatistics and Translational Medicine, Medical University of Lodz, Lodz, Poland; ^4^ Department of Pediatrics, Oncology and Hematology, Medical University of Lodz, Lodz, Poland

**Keywords:** obesity, diabetes, anti-islet autoantibodies, children, adolescents

## Abstract

**Introduction:**

One of the most important complications of obesity is insulin resistance, which leads to carbohydrate metabolism disorders such as type 2 diabetes. However, obesity is also associated with development of an autoimmune response against various organs, including pancreatic beta cells. The prevalence of such autoimmune processes in children and their possible contribution to the increased incidence of type 1 diabetes is currently unclear. Therefore, the present study assessed the prevalence of autoantibodies against pancreatic islet beta cell’s antigens in children and adolescents with simple obesity.

**Material and methods:**

This prospective observational study included pediatric patients (up to 18 years of age) with simple obesity hospitalized between 2011 and 2016 at the Department of Pediatrics, Diabetology, Endocrinology and Nephrology of the Medical University of Lodz. Children with acute or chronic conditions that might additionally affect insulin resistance or glucose metabolism were excluded. Collected clinical data included sex, age, sexual maturity ratings (Tanner`s scale), body height and weight, waist and hip circumference, amount of body fat and lean body mass. Each participant underwent a 2-hour oral glucose tolerance test with simultaneous measurements of glycaemia and insulinemia at 0`, 60` and 120`. In addition, glycated hemoglobin HbA1c, fasting and stimulated c-peptide, total cholesterol, as well as high- and low-density cholesterol and triglycerides were measured. Insulin resistance was assessed by calculating HOMA-IR index. The following autoantibodies against pancreatic islet beta cells were determined in each child: ICA - antibodies against cytoplasmic antigens of pancreatic islets, GAD - antibodies against glutamic acid decarboxylase, ZnT8 - antibodies against zinc transporter, IA2 - antibodies against tyrosine phosphatase, IAA – antibodies against insulin.

**Results:**

The study group included 161 children (57.4% boys, mean age 13.1 ± 2.9 years) with simple obesity (mean BMI z-score +2.2 ± 1.6). Among them, 28 (17.4%) were diagnosed with impaired glucose metabolism during OGTT [23 (82.2%) – isolated impaired glucose tolerance (IGT), 3 (10.7%) – isolated impaired fasting glucose (IFG), 2 (7.1%) – IFG and IGT]. Of the children tested, 28 (17.4%) were tested positive for at least one islet-specific autoantibody [with similar percentages in boys (15, 17.4%) and girls (13, 17.3%), p=0.9855], with ICA being the most common (positive in 18, 11.2%), followed by IAA (7, 4.3%), ZnT8 (5, 3.1%), GADA (3, 1.9%) and IA2 (1, 0.6%). There was no association between the presence of the tested antibodies and age, sex, stage of puberty, parameters assessing the degree of obesity, HbA1c, lipid levels and basal metabolic rate. However, autoantibody-positive subjects were more likely to present IFG or IGT in OGTT compared to those who tested completely negative (9, 32.1% vs 19, 14.3%, p=0.0280). Their HOMA-IR was also significantly higher (HOMA-IR: 4.3 ± 1.9 vs 3.4 ± 1.9, p=0.0203) and this difference remained statistically significant after adjusting for sex and age (p=0.0340).

**Conclusions:**

Children and adolescents with simple obesity presented a higher prevalence of markers of autoimmune response against pancreatic beta cells than the general population. Most often, they had only one type of antibody - ICA. The presence of autoimmune response indicators against pancreatic islet antigens is more common in obese patients with impaired carbohydrate metabolism and is associated with lower insulin sensitivity.

## Introduction

Excess body weight has become one of the most crucial challenges for pediatric healthcare. According to the World Health Organization report, the prevalence of overweight and obesity among children aged 5-19 has increased 4.5 times since 1975, meaning that in 2016, 340 million children and adolescents worldwide were overweight or obese and 124 million were obese (1). Excess body weight is associated with an increased risk of a number of disorders, the most prominent of which are disorders of carbohydrate metabolism, including diabetes (2–6). In principle, obesity is known to cause insulin resistance and secondary insulin secretion abnormalities that lead to the development of type 2 diabetes (T2D) (7). However, in children and adolescents, the impact of excess body weight might be more nuanced.

Primarily, it has been shown that the clinical course of T2D in children and adolescents differs from that in adults and exhibit a faster rate of decline in endogenous insulin secretion (8–10). Moreover, up to 1/3 of patients with T2D can be detected with the presence of at least one type of antibodies against islet antigens (11, 12). Moreover, excess body weight has been also hypothesized to contribute to the development of type 1 diabetes (T1D), the most common type of diabetes in childhood. According to the accelerator hypothesis, in individuals with HLA-dependent genetic predisposition, excess weight gain reduces insulin sensitivity, which in turn leads to pancreatic beta cells apoptosis and activation of an autoimmune response (13, 14). In addition, adipose tissue generates chronic low-grade inflammation and adversely affects the immunotolerance mechanisms (15). However, attempts to clinically verify this hypothesis have met with mixed success – the observed associations between excess body weight (birth weight, weight gain in infants and older children) and the incidence of T1D (5, 16–19) turned out to be inconclusive and largely based on selected groups of children with an underlying genetic risk for T1D.

Therefore, it is worth taking a closer look at the group of obese children who might be at increased risk not only for T2D, but also for T1D. The aim of this study was to investigate the presence of T1D-related autoimmune markers in children and adolescents with simple obesity and test whether they are associated with the development of carbohydrate metabolism disorders in those patients.

## Subjects and methods

### Ethics statement

This was a prospective observational study approved by the local Bioethics Committee of the Medical University of Lodz (No. RNN/224/15/KE) and conducted in accordance with the principles set forth in the Declaration of Helsinki

### Subjects

Between 2011-2016, we recruited children and adolescents up to 18 years of age who were routinely admitted to the Department due to obesity (defined as BMI percentile ≥95^th^ percentile) and underwent screening for glucose metabolism abnormalities. They were invited to take part in an extended panel of metabolic tests, including screening for T1D. Individuals with acute or chronic conditions that might predispose to carbohydrate metabolism disorders were excluded. We also collected data on family history of T1D and other autoimmune disorders to minimize potential bias in autoantibody prevalence due to family burden. This step was done during a follow-up period, so not all participants were available.

### Methods

Each participant underwent a comprehensive medical examination with nutritional assessment. Anthropometric parameters were measured by Harpenden stadiometer (accuracy of 0.1 cm), TANITA MC-980MA (accuracy of 0.1 kg) and non-stretchable tape (accuracy of 0, 1 cm). Body composition was analyzed by bioelectrical impedance analysis (BIA-TANITA MC-980MA), and basal metabolic rate (BMR) was calculated. Sexual maturity ratings were assessed in each child using the Tanner scale. Glycated hemoglobin (HbA1c) was measured with high-performance liquid chromatography (Bio-Rad Variant, Bio-Rad Laboratories, Hercules, USA). Lipid profile (total cholesterol, HDL-cholesterol, LDL-cholesterol, triglycerides) was performed in each child according to commonly accepted methods in the hospital laboratory.

Each participant underwent a 2-hour oral glucose tolerance test (OGTT), during which blood glucose and insulin levels were measured at 0`, 60` and 120` - then the presence of glucose metabolism disorders was determined in accordance to the ISPAD 2018 Recommendations (20). In addition, c-peptide levels (ELISA) were measured for each patient in the fasting state and 6` after stimulation with 1mg of intravenous glucagon.

Each child also underwent a comprehensive screening for pancreatic islet autoantibodies performed by the Laboratory of Immunopathology and Genetics, which is the reference laboratory for Poland, certified during the Islet AutoAntibody Standardization Program - IASP 2012-2019 (LAB604). The following autoantibodies were measured in serum (method, IASP-certified sensitivity/specificity and cut-off for positivity reported respectively):

- ICA - antibodies against cytoplasmic antigens of pancreatic islets (indirect immunofluorescence method using human pancreatic sections, 72.0% and 94.4%, cut-off: 5-10 µ. JDF depending on the substrate used),
- GAD - antibodies against glutamic acid decarboxylase (RSR ELISA method, USA, 82% and 98.9%, cut-off ≥10),
- ZnT8 - antibodies against zinc transporter (RSR ELISA method, USA, 76% and 97.8%, cut-off ≥15),
- IA2 - antibodies against tyrosine phosphatase (RSR ELISA method, USA, 70% and 95.6%, cut-off≥20),
- IA/IAA – antibodies against insulin (RIA method, UK, 42% and 100%, cut-off≥10).

### Statistical analysis

Body mass index (BMI) was calculated according to standard equation [weigh/(height in m) ^2], z-scores and percentiles were calculated based on national growth charts (21). Waist-to-height ratio (WHtR) and waist-to-hip ratio (WHT) were calculated in the standard way. Insulin resistance was assessed using HOMA-IR index (Homeostatic Model Assessment – Insulin Resistance), calculated according to the standard equation (fasting insulinemia (mU/ml) x fasting blood glucose (mmol/l)/22.5).

Distributions of continuous variables were assessed using Shapiro-Wilk test. Afterwards, tests between the groups were performed using t-test [results reported as mean± standard deviation (SD)] or Mann-Whitney`s U test (results as medians and 25-75% ranges). Qualitative variables were analyzed using the chi^2 test or fisher`s exact test for small groups.

Due to the lack of a control group, the prevalence of antibodies to pancreatic islet antigens in the study group was compared with data from the literature (22–24).

## Results

### Demographic, anthropometric, and metabolic parameters

The study group included 161 children (57.4% boys, mean age 13.1 ± 2.9 years) with simple obesity (mean BMI z-score +2.2+/0.4) (**Table 1**). Among them, 28 (17.4%) were diagnosed with impaired glucose metabolism during OGTT [23 (82.2%) – isolated impaired glucose tolerance (IGT), 3 (10.7%) – isolated impaired fasting glucose (IFG), 2 (7.1%) – IFG and IGT].

**Table 1:** Clinical and biochemical characteristics of obese children and adolescents according to the presence of beta cell autoantibodies.

	All patients (N=161)		Ab- (N=133)		Ab+ (N=28)		p-value (adj. for sex and age)
	N with available data	Mean ± SD	N with available data	Mean ± SD	N with available data	Mean ± SD	
Age [years]	161	13.1 ± 5.8	133	13.0 ± 2.9	28	13.8 ± 2.8	0.1840
BMI z-score	161	2.2 ± 1.6	133	2.2 ± 0.4	28	2.3 ± 0.4	0.7382
Waist (cm)	109	95.9 ± 72.0	89	95.3 ± 10.5	20	98.6 ± 11.5	0.2271 (0.6896)
Hips (cm)	107	102.8 ± 78.0	89	102.1 ± 11.6	18	106.5 ± 13.8	0.1590 (0.6929)
WHR	106	0.9 ± 0.7	88	0.9 ± 0.1	18	0.9 ± 0.1	0.7959 (0.2889)
WHtR	96	0.6 ± 0.5	78	0.59 ± 0.1	18	0.63 ± 0.03	0.0739 (0.0471)
Body composition, fat percentage [%]	131	38.1 ± 23.9	112	38.2 ± 6.7	19	37.4 ± 6.3	0.6193 (0.6449)
BMR [kJ]	131	7 563.5 ± 4 996.0	112	7513.6 ± 1464.6	19	7857.4 ± 1345.7	0.3406 (0.9558)
OGTT glycemia 0’	161	83.6 ± 59.0	133	83.2 ± 8.2	28	85.9 ± 10.3	0.1297
OGTT glycemia 60’	161	133.1 ± 78.6	133	131.0 ± 27.2	28	143.3 ± 31.5	0.0360
OGTT glycemia 120’	161	117.6 ± 63.0	133	117.4 ± 21.3	28	118.6 ± 24.9	0.7926
OGTT insulin 0’	108	18.0 ± 1.0	87	17.1 ± 8.7	21	21.3 ± 8.6	0.0496
OGTT insulin 60’	106	127.0 ± 3.0	85	105.7 ± 74.4	21	213.4 ± 226.8	0.0004
OGTT insulin 120’	107	104.3 ± 9.9	87	96.0 ± 70.2	20	140.5 ± 144.2	0.0447
C-peptide – fasting [ng/ml]	150	3.1 ± 1.1	122	3.0 ± 1.2	28	3.4 ± 1.2	0.1420
C-peptide – stimulated [ng/ml]	145	7.8 ± 2.3	120	7.6 ± 2.8	25	8.7 ± 3.7	0.0925
C-peptide – ratio after stimulation	143	2.7 ± 0.6	118	2.7 ± 0.9	25	2.7 ± 0.9	0.9709
HOMA-IR	158	3.5 ± 0.2	132	3.4 ± 1.9	26	4.3 ± 1.9	0.0203 (0.0340)
HbA1c [%]	159	5.4 ± 4.4	131	5.4 ± 0.3	28	5.5 ± 0.3	0.3111
cholesterol [mg/dl]	109	169.5 ± 99.0	87	171.4 ± 34.2	22	162.2 ± 35.7	0.2644
TG [mg/dl]	109	126.4 ± 32.7	87	130.3 ± 70.6	22	110.8 ± 62.9	0.2409
HDL [mg/dl]	110	45.0 ± 26.6	88	45.3 ± 9.9	22	43.8 ± 10.8	0.5540
LDL [mg/dl]	108	105.6 ± 8.9	86	106.1 ± 33.5	22	103.5 ± 28.5	0.7410

(BMI, body mass index; WHR, waist to hip ratio; WHtR, waist to height ratio; BMR, basal metabolic rate; OGTT, oral glucose tolerance test; HOMA, IR, Homeostatic Model Assessment – Insulin Resistance; BMR, basal metabolic rate HDL, cholesterol, high, density lipoprotein, cholesterol; LDL, cholesterol, low, density lipoprotein, cholesterol).

### The prevalence of anti-islet antibodies

Of the children tested, 28 (17.4%) were positive for at least one islet-specific autoantibody [with similar percentages in boys (15, 17.4%) and girls (13, 17.3%), p=0.9855], with ICA being the most common (positive in 18, 11.2%), followed by IAA (7, 4.3%), ZnT8 (5, 3.1%), GAD (3, 1.9%) and IA2 (1, 0.6%). The prevalence of positive results for most autoantibodies in the study group was similar to general population (historic reference for IAA – 4%, anti-GAD – 2%, IA2 – 0.8%, ZnT8 -2%, all p>0.05). However, we noted that obese children were more often positive for at least one autoantibody (17.3% vs 4.9%, p<0.0001) and particularly for ICA autoantibodies (11.2% vs historical reference of 3%, p<0.0001).

### Association of the autoimmune response against pancreatic islets with participants` clinical characteristics

Children positive for at least one autoantibody were non-significantly older (13.8 ± 2.8 vs 13.0 ± 2.9, p=0.1840) than those who tested negative and presented similar sex maturity ratings (median 3 (25-75%: 2 to 5) vs 3 (25-75%: 2 to 5), p= 0.4135), HbA1c (5.4 ± 0.3 vs 5.3 ± 0.3, p=0.3111), c-peptide (fasting: 3.4 ± 1.2 vs 3.0 ± 1.2, p=0.1420; stimulated: 8,7 ± 3,7 vs 7,6 ± 2,8, p=0.0925) and lipid profile. They were also comparable in terms of BMI z-scores, body fat percentage and estimated Basal Metabolic Rate. However, autoantibody-positive subjects had a higher waist to height ratio (WHtR: 0,63 ± 0,03 vs 0,59 ± 0,1, p=0.047 - adj. for sex and age).

### The relationship between the occurrence of an autoimmune reaction against pancreatic islets and the carbohydrate metabolism disorders

**Figure 1:**
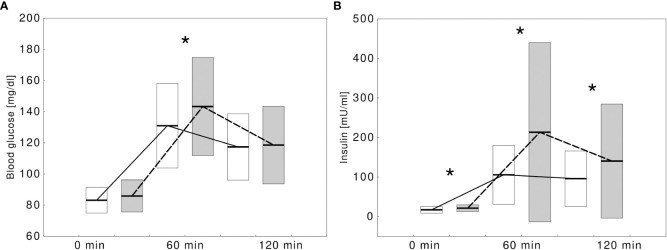
Comparison of blood glucose **(A)** and insulin **(B)** concentrations during oral glucose tolerance test in obese children without type 1 diabetes-specific autoantibodies (white boxes, solid line) and with at least one autoantibody (gray boxes, dashed lines). Significant differences (p<0.05) were marked with an asterix (*).

Children with at least one positive autoantibody were more likely to present IFG or IGT in OGTT compared with those who tested completely negative (9, 32.1% vs 19, 14.3%, p=0.0280). The autoantibody-positive children showed significantly higher glycemia at 60 min. of OGTT and higher insulinemia at all three time points of OGTT (**Figure 1**). Their HOMA-IR index was significantly higher (HOMA-IR: 4.3 ± 1.9 vs 3.4 ± 1.9, p=0.0203) and the difference remained statistically significant after adjusting for sex and age (p=0.0340). A detailed comparison between the groups is presented in **Table 1**.

### Association of autoimmune response against pancreatic islets with family history of type 1 diabetes and other autoimmune diseases

In 54 (33.5%) children, a family history of type 1 diabetes or other autoimmune diseases were unavailable. Among the 107 children with a complete family history available, none of the 16 autoantibody-positive children (0%) had any known relatives with type 1 diabetes, compared with 7 autoantibody-negative children (7.7%), which was likely due to chance (p=0.5910). Similarly, both autoantibody-positive and autoantibody-negative children had a similar prevalence of type 1 diabetes or other autoimmune diseases in first degree relatives (autoantibody-positive: 2 (12.5%) vs autoantibody-negative: 15 (16/5%), p=1.0000)

### Patients with multiple anti-islet antibodies

Out of autoantibody-positive children, four (2.5%) were positive for at least two autoantibodies, marking pre-clinical stages of type 1 diabetes – their exact titers and patients` clinical features are included in **Table 2**. Over the following seven years, only one subject developed clinically evident type 1 diabetes as an adult (more data unavailable).

**Table 2:** Characteristics of obese children and adolescents with multiple beta cell autoantibodies (≥ 2Ab).

Patient ID		1	2	3	4
Autoantibodies and cut-offs	ICA (>0)	0	**20**	**40**	**40**
	anti-GAD (>=10)	0.73	**17**	**131.14**	**131.1**
	anti-IA2 (>=20)	5.24	1.33	10.26	0
	ZnT8 (>=15)	**22.06**	3.29	**16.17**	0.8
	IAA (>=10)	**23.14**	3.41	**41.96**	1.58
Clinical characteristics	Age	13.05	12.47	16.68	17.23
	Sex	F	F	M	M
	Family history	N/A	N/A	Negative	Negative
	Fasting c-peptide	3.16	3.07	5.57	2.3
	HbA1c	5.2	5.3	5.2	5.5
	HOMA-IR	4.3	4.5	3.9	6.34
	BMR	7510	8117	9912	8118
	Fasting blood glucose	79	96	82	82
	Blood glucose – 120` of OGTT	130	81	**169**	118

(ICA, antibodies against cytoplasmic antigens of pancreatic islets; GAD, antibodies against glutamic acid decarboxylase; ZnT8, antibodies against zinc transporter; IA2, antibodies against tyrosine phosphatase; IAA, anti-insulin antibodies; HOMA-IR- Homeostatic Model Assessment – Insulin Resistance; BMR-basal metabolic rate).

## Discussion

Our most important observation is that the prevalence of at least one islet-specific autoantibody in a sample of children and adolescents with simple obesity was as much as 3.5 times higher than in general population. The prevalence of islet autoantibodies is well-established in patients with T1D or at increased risk of developing the disease (first degree relationship to the patient, genetic screening) (24, 25).

In contrast, reports on the frequency of autoimmune anti-islet responses in people with excess body weight or with T2D are less numerous and primarily relate to adults. About 2-12% of adults with clinically diagnosed T2D were found to have detectable levels of antibodies to pancreatic islet antigens in peripheral blood (26–28). It was also suggested that the degree of obesity might be associated with the risk of developing islet autoantibodies in adults with T2D - among 204 patients with T2D and excess body weight, the presence of at least one of the three antibodies (GADA, IA2, and ZnT8A) was found in 6.4% of all subjects and in as many as 12.8% when considering those with severe obesity (BMI > = 40 kg/m2) (29). On the other hand, studies on pediatric patients with T2D reported the presence of at least one anti-islet antibody in approximately 30% of them (11, 30–32). The presence of at least one anti-islet antibody (GADA or IA2) in obese children with T2D was associated with younger age, lower insulin secretion, higher HbA1c, and a higher incidence of diabetic ketoacidosis at diagnosis (33). Our estimated prevalence of positive autoantibodies was, as expected, lower than that observed in children and adolescents with T2D.

Relatively few papers have examined the anti-islet autoimmune response in obese children and adolescents without previously diagnosed diabetes. Cambuli et al. assessing the presence of anti-islet antibodies (GADA, IA2, IAA) in 686 overweight/obese children and adolescents (mean age 10.3 ± 3.2 years) found the presence of at least one anti-islet antibody in 2.18% of the subjects (2 antibodies in 0.7% and 1 antibody in 0.15%) and this value did not differ significantly from the result obtained in the control group with normal body weight (1.8%) (34). GADA-1.89% was the most frequently detected, followed by IA2-0.87% and IAA-0.43%. The apparent discrepancy with our observed prevalence results from including ICA in our study (and to a lesser extent, ZnT8). In our study of obese children and adolescents, ICA was the most common anti-islet antibody, its prevalence significantly increased compared to literature reports for general population (22–24). ICA antibodies were the first identified antibodies against pancreatic islet antigens and were identified using indirect immunofluorescence on frozen sections of human pancreas with blood group 0. The demonstration of ICA by indirect immunofluorescence is an illustration of general islet autoimmunization and identifies several beta-cell antigens simultaneously (24). The finding of isolated positive ICA despite negative GADA, IA-2A, IAA and ZnT8A results in children with newly diagnosed T1D indicates that ICA testing detects a wide range of islet-specific antibodies and sometimes cross-reacting antibodies in serum samples (35). Pollannen et al. in the Finnish population-based Diabetes Prediction and Prevention (DIPP) type 1 study, which aims to monitor the emergence of anti-islet autoantibodies in children with increased HLA-dependent susceptibility to T1D, showed high sensitivity of ICA in identifying clinical disease progression, but unlike previously published 5-year follow-up data, lower specificity than other autoantibodies tested by biochemical methods (IAA, GADA, IA2) (36). It should also be kept in mind that the inconclusive results of the work on the predictive value of ICA for the development of T1D may be due to the fact that ICA determination is the most operator-dependent test of all the tests measuring the level of anti-islet autoantibodies (23, 37). In our study, the determination of all types of antibodies against pancreatic islet antigens was performed in a reference laboratory for Poland, certified during the Islet AutoAntibody Standardization Program - IASP 2012-2019.

We also need to comment on the patients in whom we observed multiple positive islet autoantibodies, although the numbers was too low for statistical analysis. It is estimated that 70% of patients who have at least 2 types of anti-islet antibodies will develop clinical type 1 diabetes mellitus within 10 years (38). Our study identified 4 individuals with at least 2 anti-islet antibodies, one of them was also diagnosed with dysglycemia (IGT). According to the ADA criteria, they were individuals with type 1 diabetes, 3 individuals in the first stage (autoimmunity), and one individual in the second stage (autoimmunity and dysglycemia) (39). Only for the latter individual, follow-up data were available - after 7 years since testing, he was clinically diagnosed with T1D (stage III) and insulin therapy had to be implemented.

In the classical view, decreased insulin sensitivity caused by excess adipose tissue heralds the progression of metabolic disorders towards pre-diabetes (IFG, IGT) and then diabetes (40, 41). In the present study, impaired glucose metabolism in the form of IFG and/or IGT was found in 28 (17.4%) obese children and adolescents (none of the subjects were diagnosed with diabetes). In comparison, studies from European countries estimate the incidence of IFG/IGT in obese children and adolescents at 5.7-17.1%, and type 2 diabetes at 1.4% (42, 43). The observed differences may be related to the methodology of studies (age of subjects, definition of obesity) and the influence of such recognized risk factors as e.g., eating habits, physical activity, genetic conditions, or socioeconomic factors (40). A prospective study by Galderisi et al. involving a multi-ethnic group of 526 obese adolescents (10.6-14.2 years of age) showed the transient nature of IGT - after 2 years, 65% of respondents returned from IGT to normal glucose tolerance (NGT), 27% maintained IGT and 8% developed T2D (44). Unfortunately, long-term follow-up data were not collected in our study to ascertain the future clinical, metabolic and immune status of the patients (except for the one already-mentioned case).

In the study by Cambuli et al. in children with excess body weight, impaired glucose metabolism were identified in 11.37% of subjects (IFG-8.16%, IGT-3.2%, diabetes-0.6%), and individuals with positive antibodies characterized by a higher incidence of pre-diabetes or diabetes mellitus and higher glycaemia at 120 min. OGTT than those without antibodies (27% vs 11% and 133 mg/dL vs 105.4 mg/dL, respectively) (34). This was consistent with our observation. HbA1c, however, was comparable in both subgroups.

We also investigated the possible relationship between the presence of autoimmune markers and the degree of obesity. The effect of type of obesity was not assessed, as all study participants were diagnosed with abdominal obesity. In our group, neither BMI z-score nor total body fat was related to the presence of islet autoantibodies. While BMI and body fat are good indicators of obesity, it was mainly the increase in visceral adipose tissue that was associated with insulin resistance and low-grade chronic inflammation, contributing not only to impaired insulin function and impaired insulin secretion, but also to the promotion of autoimmune responses against various organs (40, 45–49). We could not reliably measure or estimate that characteristics in this study setting. However, we noted that seropositive patients were characterized by higher WHtR, which corresponds to the severity of abdominal obesity, as well as higher insulin resistance, consistent with reports that abdominal obesity and insulin resistance have a negative effect on the development of an autoimmune response against pancreatic islet antigens (47, 48). According to a study in overweight/obese adults with T2D by Philla et al., the presence of antibodies was associated with lower resting C-peptide levels (29). We did not find a similar relationship in obese children and adolescents, either in terms of fasting or stimulated C-peptide secretion. Similarly, we found no significant differences in the lipid profiles reported in adults (islet autoantibodies associated with higher serum HDL-cholesterol concentration) (29).

The pubertal pediatric population has the physiologically lowest insulin sensitivity and the highest incidence of type 2 diabetes (50, 51). Girls are more likely to develop type 2 diabetes than boys, which may be due to overstimulation of the insulin receptor in pancreatic beta cells by endogenous estrogen (52). The pubertal period is also associated with a higher incidence of T1D than the early school age (53). In the present study, there was no correlation between age, gender, and pubertal stage in obese children and adolescents and the occurrence of anti-islet autoimmunity.

Genetic factors are crucial in the development of both T1D and T2D, and a burdensome family history and/or the presence of a genetically determined susceptibility to develop diabetes may be the determining factors tin bringing children and adolescents under close clinical observation and laboratory screening (36, 51). In the present study, data on the presence of T1D and other autoimmune diseases in 1st and 2nd degree relatives were obtained in 2/3 of patients. Obese children with anti-islet autoimmunity did not differ significantly in terms of family burden of autoimmune diseases, including T1D, from children without autoantibodies. It should be added that one patient diagnosed with T1D stage II according to ADA criteria had no family history of T1D or other autoimmune diseases.

There are several limitations of this work that should be disclosed. First, this was a single-center study, and patients were assessed at a single visit, without subsequent follow-up for autoimmunity markers or progression towards diabetes (except in one case). There was also no healthy control group available, which forced us to use literature-based data on the islet-autoantibody prevalence as reference. Finally, the analysis did not consider the effect of genetic HLA-dependent and HLA-independent susceptibility on the development of diabetes, and in some cases, it was not possible to verify information on the family burden of diabetes in the subjects.

On the other hand, we provided data from a fairly large sample of children and adolescents with simple obesity. The patients were of the same ethnic origin and were not pre-selected and matched in terms of genetic predisposition to develop diabetes. Anthropometric parameters were interpreted using national percentile grids, and both the effect of subjects’ age and pubertal stage were included in the data analysis. Five types of antibodies to pancreatic islet antigens were tested, and all tests were performed at a single reference center. Not only the frequency of carbohydrate metabolism disorders was analyzed, but also parameters determining pathogenetic phenomena related to glucose homeostasis - insulin sensitivity (HOMA-IR) and insulin secretion (OGTT, C-peptide - fasting and glucagon stimulated) were examined.

## Conclusions

Children and adolescents with simple obesity in this study presented significantly higher rates of being positive for T1D-related islet autoantibodies, however, most of the difference was due to an increased prevalence of positive ICA. The presence of autoimmune markers was associated with insulin resistance and risk of prediabetes. Our results support that hypothesis that obesity might play a role in the development of T1D, but further research is needed. It might be worthwhile to extend diabetes screening in obese children and adolescents to include monitoring of the autoimmune response against pancreatic beta cells.

## Data availability statement

The raw data supporting the conclusions of this article will be made available by the authors, without undue reservation.

## Ethics statement

The studies involving human participants were reviewed and approved by the Bioethics Committee of the Medical University of Lodz (No. RNN/224/15/KE). Written informed consent to participate in this study was provided by the participants’ legal guardian/next of kin.

## Author contributions

AC-F conceived the study, contributed to the study design, data collection, analysis, and drafting of the manuscript, IP contributed to the study design, data collection, analysis and wrote the first draft of the manuscript. AM contributed to the study design, data collection, analysis, and drafting of the manuscript, KW contributed to the analysis and drafting of the manuscript, AS conceived the study, contributed to the study design, analysis and drafting of the manuscript. All authors contributed to the article and approved the submitted version.

## References

[1] Obesity and overweight. Available at: https://www.who.int/news-room/fact-sheet/detail/obesity-and-overweight (Accessed 15 September 2022).

[2] Ng M Fleming T Robinson M Thomson B Graetz N Margono C. (The GBD 2013 obesity collaboration) global, regional and national prevalence of overweight and obesity in children and adults 1980-2013: A systematic analysis lancet. Lancet (2014) 384(9945):766–81. doi: 10.1016/S0140-6736(14)60460-8 PMC4624264 24880830

[3] Faienza MF Wang DQ Frühbeck G Garruti G Portincasa P. The dangerous link between childhood and adulthood predictors of obesity and metabolic syndrome. Intern Emerg Med (2016) 11:175–82. doi: 10.1007/s11739-015-1382-6 26758061

[4] Lindberg L Danielsson P Persson M Marcus C Hagman E. Association of childhood obesity with risk of early all-cause and cause-specific mortality: A Swedish prospective cohort study. PloS Med (2020) 17:e1003078. doi: 10.1371/journal.pmed.1003078 32187177 PMC7080224

[5] Nucci AM Virtanen SM Cuthbertson D Ludvigsson J Einberg U Huot C. TRIGR investigators growth and development of islet autoimmunity and type 1 diabetes in children genetically at risk. Diabetologia (2021) 64:826–35. doi: 10.1007/s00125-020-05358-3 PMC7940594 33474583

[6] Obita G Alkhatib A. Disparities in the prevalence of childhood obesity-related comorbidities: A systematic review front public health. Front Public Health (2022) 10:923744. doi: 10.3389/fpubh.2022.923744 35874993 PMC9298527

[7] Tuomi T Santoro N Caprio S Cai M Weng J Groop L. The many faces of diabetes: a disease with increasing heterogeneity. Lancet (2014) 383:1084–94. doi: 10.1016/S0140-6736(13)62219-9 24315621

[8] Wilmot E Idris I. Early onset type 2 diabetes: risk factors, clinical impact and management. Ther Adv Chronic Dis (2014) 5:234–44. doi: 10.1177/2040622314548679 PMC4205573 25364491

[9] Dabelea D Mayer-Davis EJ Andrews JS Dolan LM Pihoker C Hamman RF. Clinical evolution of beta cell function in youth with diabetes: the SEARCH for diabetes in youth study. Diabetologia (2012) 55:3359–68. doi: 10.1007/s00125-012-2719-6 PMC4492685 22990715

[10] Hannon TS Arslanian SA. The changing face of diabetes in youth: lessons learned from studies of type 2 diabetes. Ann N Y Acad Sci (2015) 1353:113–37. doi: 10.1111/nyas.12939 26448515

[11] Umpaichitra V Banerji MA Castells S. Autoantibodies in children with type 2 diabetes mellitus. J Pediatr Endocrinol Metab (2002) 15 Suppl 1:525–30. 12017227

[12] Reinehr T Schober E Wiegand S Thon A Holl R DPV-Wiss Study Group. Beta-cell autoantibodies in children with type 2 diabetes mellitus: subgroup or misclassification? Arch Dis Child (2006) 91:473–7. doi: 10.1136/adc.2005.088229 PMC2082766 16449253

[13] Wilkin TJ. The accelerator hypothesis: weight gain as the missing link between type I and type II diabetes. Diabetologia (2001) 44:914–22. doi: 10.1007/s001250100548 11508279

[14] Knerr I Wolf J Reinehr T Stachow R Grabert M Schober E. The a’ccelerator hypothesis’: relationship between weight, height, body mass index and age at diagnosis in a large cohort of 9,248 German and Austrian children with type 1 diabetes mellitus. Diabetologia (2005) 48:2501–2504. doi: 10.1007/s00125-005-0033-2 16283240

[15] Tsigalou C Vallianou N Dalamaga M. Autoantibody production in obesity: Is there evidence for a link between obesity and autoimmunity? Curr Obes Rep (2020) 9:245–54. doi: 10.1007/s13679-020-00397-8 32632847

[16] Lamb MM Yin X Zerbe GO Klingensmith GJ Dabelea D Fingerlin TE. Height growth velocity, islet autoimmunity and type 1 diabetes development: the diabetes autoimmunity study in the young diabetologia. Diabetologia (2009) 52:2064–71. doi: 10.1007/s00125-009-1428-2 PMC2813468 19547949

[17] Verbeeten KC Elks CE Daneman D Ong KK. Association between childhood obesity and subsequent type 1 diabetes: A systematic review and meta-analysis. Diabetes Med (2011) 28:10–8. doi: 10.1111/j.1464-5491.2010.03160.x 21166841

[18] Larsson HE Vehik K Haller MJ Liu X Akolkar B Hagopian W. Growth and risk for islet autoimmunity and progression to type 1 diabetes in early childhood: The environmental determinants of diabetes in the young study. Diabetes (2016) 65:1988–95. doi: 10.2337/db15-1180 PMC4915577 26993064

[19] Ferrara-Cook C Geyer SM Evans-Molina C Libman IM Becker DJ Gitelman SE. Type 1 diabetes TrialNet study group excess BMI accelerates islet autoimmunity in older children and adolescents. Diabetes Care (2020) 43:580–7. doi: 10.2337/dc19-1167 PMC7035590 31937610

[20] Mayer-Davis EJ Kahkoska AR Jefferies C Dabelea D Balde N Gong CX. ISPAD clinical practice consensus guidelines 2018: Definition, epidemiology, diagnosis and classification of diabetes in children and adolescents. Pediatr Diabetes (2018) Suppl 27:7–19. doi: 10.1111/pedi.12773 PMC7521365 30226024

[21] Kułaga Z Różdżyńska-Świątkowska A Grajda A Gurzkowska B Wojtyło M Góźdź M. Percentile charts for growth and nutritional status assessment in polish children and adolescents from birth to 18 year of age standardy Medyczne/Pediatria. Standardy Medyczne/Pediatria (2015) 12:119–35.

[22] Smeets S Diedert Luc De Paep DL Stangé G Verhaeghen K van der Auwera B Keymeulen B. Insulitis in the pancreas of non-diabetic organ donors under age 25 years with multiple circulating autoantibodies against islet cell antigens. Virchows Arch (2021) 479:295–304. doi: 10.1007/s00428-021-03055-z 33594586 PMC8364522

[23] Velluzzi F Secci G Sepe V Klersy C Shattock M Foxon R. Prediction of type 1 diabetes in sardinian schoolchildren using islet cell autoantibodies: 10-year follow-up of the sardinian schoolchildren type 1 diabetes prediction study. Acta Diabetol (2016) 53:73–9. doi: 10.1007/s00592-015-0751-y 25896008

[24] Franke B Galloway TS Wilkin TJ. Developments in the prediction of type 1 diabetes mellitus, with special reference to insulin autoantibodies. Diabetes Metab Res Rev (2005) 2:395–415. doi: 10.1002/dmrr.554 15895384

[25] Ross C Ward ZJ Gomber A Owais M Yeh YM Reddy CL. The prevalence of islet autoantibodies in children and adolescents with type 1 diabetes mellitus: A global scoping review. Front Endocrinol (Lausanne) (2022) 13:815703. doi: 10.3389/fendo.2022.815703 35185797 PMC8851309

[26] Davis TME Wright AD Mehta ZM Cull CA Stratton IM Bottazzo GF. Islet autoantibodies in clinically diagnosed type 2 diabetes: prevalence and relationship with metabolic control (UKPDS 70). Diabetologia (2005) 48:695–702. doi: 10.1007/s00125-005-1690-x 15729570

[27] Zinman B Kahn SE Haffner SM O'Neill MC Heise MA Freed MI. Phenotypic characteristics of GAD antibody-positive recently diagnosed patients with type 2 diabetes in north America and Europe. Diabetes (2004) 53:3193–200. doi: 10.2337/diabetes.53.12.3193 15561950

[28] Trabucchi A Faccinetti NI Guerra LL Puchulu FM Frechtel GD Poskus E. Detection and characterization of ZnT8 autoantibodies could help to screen latent autoimmune diabetes in adult-onset patients with type 2 phenotype. Autoimmunity (2012) 45:137–42. doi: 10.3109/08916934.2011.604658 21875382

[29] Pilla SJ Balasubramanyam A Knowler WC Lazo M Nathan DM Pi-Sunyer X. Islet autoantibody positivity in overweight and obese adults with type 2. Diabetes Autoimmun (2018) 51:408–16. doi: 10.1080/08916934.2018.1547711 PMC6362362 30661481

[30] Reinehr T Schober E Wiegand S Thon A Holl R on behalf of the DPV-Wiss Study Group. β-cell autoantibodies in children with type 2 diabetes mellitus: subgroup or misclassification? Arch Dis Child (2006) 91:473–7. doi: 10.1136/adc.2005.088229 PMC2082766 16449253

[31] Alyafei F Soliman A Alkhalaf F Sabt A De Sanctis V Elsayed N. Prevalence of β-cell antibodies and associated autoimmune diseases in children and adolescents with type 1 diabetes (T1DM) versus type 2 diabetes (T2DM) in Qatar. Acta BioMed (2018) 89(S5):32–9. doi: 10.23750/abm.v89iS4.7359 PMC6179090 30049930

[32] Tfayli H Bacha F Gungor N Arslanian S. Phenotypic type 2 diabetes in obese youth: secretion in islet cell antibody-negative versus -positive patients. Diabetes (2009) 58:738–44. doi: 10.2337/db08-1372 PMC2646074 19073767

[33] Rivera-Vega MY Flint A Winger DG Libman I Silva Arslanian S. Obesity and youth diabetes: Distinguishing characteristics between islet cell antibody positive vs. negative patients over time. Pediatr Diabetes (2015) 16:375–81. doi: 10.1111/pedi.12249 PMC4457715 25482141

[34] Cambuli VM Incani M Cossu E Congiu T Scano F Pilia S. Prevalence of type 1 diabetes autoantibodies (GADA, IA2, and IAA) in overweight and obese children. Diabetes Care (2010) 33:820–2. doi: 10.2337/dc09-1573 PMC2845034 20040655

[35] Andersson C Kolmodin M Ivarsson S-A Carlsson A Forsander G Lindblad B. Islet cell antibodies (ICA) identify autoimmunity in children with new onset diabetes mellitus negative for other islet cell antibodies. Pediatr Diabetes (2014) 15:336–44. doi: 10.1111/pedi.12093 24206368

[36] Pöllänen PM Ryhänen SJ Toppari J Ilonen J Vähäsalo P Veijola R. Dynamics of islet autoantibodies during prospective follow-up from birth to age 15 years. J Clin Endocrinol Metab (2020) 105:e4638–51. doi: 10.1210/clinem/dgaa624 PMC7686032 32882033

[37] Bonifacio E Achenbach P. Birth and coming of age of islet autoantibodies. Clin Exp Immunol (2019) 198:294–305. doi: 10.1111/cel.13360 31397889 PMC6857083

[38] Ziegler AG Rewers M Simell O Simell T Lempainen J Steck A. Seroconversion to multiple islet autoantibodies and risk of progression to diabetes in children. JAMA (2013) 309:2473–9. doi: 10.1001/jama.2013.6285 PMC4878912 23780460

[39] Insel RA Dunne JL Atkinson MA Chiang JL Dabelea D Gottlieb PA. Staging presymptomatic type 1 diabetes: a scientific statement of JDRF, the endocrine society, and the American diabetes association. Diabetes Care (2015) 38:1964–74. doi: 10.2337/dc15-1419 PMC5321245 26404926

[40] Valaiyapathi B Gower B Ashraf AP. Pathophysiology of type 2 diabetes in children and adolescents. Curr Diabetes Rev (2020) 16:220–9. doi: 10.2174/1573399814666180608074510 PMC7516333 29879890

[41] Weiss R Magge SN Santoro N Giannini C Boston R Holder T. Glucose effectiveness in obese children: Relation to degree of obesity and dysglycemia. Diabetes Care (2015) 38:689–95. doi: 10.2337/dc14-2183 PMC4370330 25633663

[42] Hagman E Reinehr T Kowalski J Ekbom A Marcus C Holl RW. Impaired fasting glucose prevalence in two nationwide cohorts of obese children and adolescents. Int J Obes (Lond) (2014) 38:40–5. doi: 10.1038/ijo.2013.124 PMC3884136 23828099

[43] Koutny F Weghuber D Bollow E Greber-Platzer S Hartmann K Körner A. Prevalence of prediabetes and type 2 diabetes in children with obesity and increased transaminases in European German-speaking countries. Anal APV initiative Pediatr Obes (2020) 15:e12601. doi: 10.1111/ijpo.12601 PMC7079233 31810110

[44] Galderisi A Giannini C Weiss R Kim G Shabanova V Santoro N. Trajectories of changes in glucose tolerance in a multiethnic cohort of obese youths: an observational prospective analysis. Lancet Child Adolesc Health (2018) 2:726–35. doi: 10.1016/S2352-4642(18)30235-9 PMC6190831 30236381

[45] González N Moreno-Villegas Z González-Bris A Egido J Lorenzo O. Regulation of visceral and epicardial adipose tissue for preventing cardiovascular injuries associated to obesity and diabetes. Cardiovasc Diabetol (2017) 16:44. doi: 10.1186/s12933-017-0528-4 28376896 PMC5379721

[46] Hershkop K Besor O Santoro N Pierpont B Caprio S Weiss R. Adipose insulin resistance in obese adolescents across the spectrum of glucose tolerance. J Clin Endocrinol Metab (2016) 101:2423–31. doi: 10.1210/jc.2016-1376 PMC4891802 27054297

[47] Versini M Jeandel PY Rosenthal E Shoenfeld Y. Obesity in autoimmune diseases: not a passive bystander. Autoimmun Rev (2014) 13:981–1000. doi: 10.1016/j.autrev.2014.07.001 25092612

[48] Tiberti C Zampetti S Capoccia D Campagna G Lucantoni F Anastasi E. Evidence of diabetes-specific autoimmunity in obese subjects with normal glucose tolerance. Diabetes Metab Res Rev (2018) 8 20:e3055. doi: 10.1002/dmrr.3055 30129269

[49] Brooks-Worrell BM Palmer JP. Setting the stage for islet autoimmunity in type 2 diabetes: Obesity-associated chronic systemic inflammation and endoplasmic reticulum (ER) stress. Diabetes Care (2019) 42:2338–46. doi: 10.2337/dc19-0475 PMC7364670 31748213

[50] Zeitler P Arslanian S Fu J Pinhas-Hamiel O Reinehr T Tandon N. ISPAD clinical practice consensus guidelines 2018 type 2 diabetes mellitus in youth. Pediatr Diabetes (2018) Suppl 27:28–46. doi: 10.1111/pedi.12719 29999228

[51] Copeland KC Zeitler P Geffner M Guandalini C Higgins J Hirst K. Characteristics of adolescents and youth with recent-onset type 2 diabetes: the TODAY cohort at baseline. J Clin Endocrinol Metab (2011) 96:159–67. doi: 10.1210/jc.2010-1642 PMC3038479 20962021

[52] Nadal A Alonso-Magdalena P Soriano S Quesada I Ropero AB. The pancreatic beta-cell as a target of estrogens and xenoestrogens: Implications for blood glucose homeostasis and diabetes. Mol Cell Endocrinol (2009) 304:63–8. doi: 10.1016/j.mce.2009.02.016 19433249

[53] Norris JM Johnson RK Stene LC. Type 1 diabetes–early life origins and changing epidemiology. Lancet Diabetes Endocrinol (2020) 8:226–38. doi: 10.1016/S2213-8587(19)30412-7 PMC7332108 31999944

